# Vitamin D Deficiency is Associated with Overweight and/or Obesity among Schoolchildren in Central Ethiopia: A Cross-Sectional Study

**DOI:** 10.3390/nu8040190

**Published:** 2016-04-01

**Authors:** Tolassa Wakayo, Susan J. Whiting, Tefera Belachew

**Affiliations:** 1 College of Health Sciences, Jimma University, Jimma, Ethiopia; teferabelachew@gmail.com; 2 College of Pharmacy and Nutrition, University of Saskatchewan, Saskatoon, SK S7N 2Z4, Canada; susan.whiting@usask.ca

**Keywords:** vitamin D deficiency, overweight/obesity, schoolchildren, Ethiopia

## Abstract

Childhood and adolescent obesity is an international public health problem leading to an increased risk of adulthood obesity, mortality and morbidity. Its prevalence is increasing in low-income populations, and we hypothesized it may be associated with vitamin D deficiency. Low vitamin D status is a worldwide public health issue including in Ethiopia; however, no one has examined overweight/obesity in Ethiopian schoolchildren with regard to vitamin D status. The Analyses of a data set from a school-based cross-sectional study conducted in Adama Town (*n* = 89) and in rural Adama Woreda (*n* = 85) was carried out to determine vitamin D deficiency and its association with overweight and/or obesity. Data on a total of 174 schoolchildren aged 11–18 years was used for these analyses. The overall prevalence of overweight and/or obesity was 10.3%, with 8.5% overweight and 2.3% obese; the prevalence of underweight was 19%. In the multivariable logistic regression model, vitamin D deficiency, being in the higher age group, female sex and urban residence of students, their mothers’ occupation of being employed and their households’ high and middle socioeconomic status were significantly associated with overweight and/or obesity. We concluded that vitamin D deficiency is an independent predictor significantly associated with overweight and/or obesity among schoolchildren from rural and urban settings in Ethiopia. The results imply the need for behavior change communications on the importance of exposure to sunlight to produce adequate vitamin D to curb this emerging health problem of overweight/obesity following economic growth and globalization in Ethiopia. As this study only highlighted the association, prospective studies and randomized controlled trials are required to establish causality.

## 1. Introduction

Childhood and adolescence obesity is an international public health problem leading to an increased risk of adult obesity and associated mortality and morbidity [1]. Although it has been previously associated primarily with high-income countries, overweight and obesity are now becoming prevalent in populations from low- and middle-income countries due to the effect of globalization [2]. While the prevalence of obesity may be stabilizing in developed countries [3,4,5], it is now increasing in developing countries [6,7,8,9]. This change is currently high in Middle East and Asian countries [10,11], but Africa is also experiencing a shift from underweight to overweight. In Africa, there are socioeconomic, nutritional and physical activity transitions taking place, which these lead to increases in the use of energy-saving devices, the availability of cheap high-calorie dense foods and limited participation in physical activity at home and at school [12,13,14,15,16,17]. The trend of rising overweight/obesity prevalence has been observed in Ethiopia, particularly in women [18] and children [19]. The fact that Ethiopia is one of the countries in East Africa undergoing rapid socioeconomic growth in the recent years is assumed to be a risk factor for this rising trend in overweight and/or obesity given that overweight and/or obesity is directly related to socioeconomic status in developing economies [20].

Vitamin D deficiency has also been identified as a worldwide public health issue that is associated with an increase in the prevalence of related-chronic diseases. Vitamin D is produced from cholesterol in the skin upon exposure to sunlight. It can also be obtained from foods and supplements. Apart from being needed for bone health and calcium homeostasis, vitamin D has extra-skeletal functions [21]. *In vitro* and animal studies over the past decades have extended the role of vitamin D to other tissues, including the pancreas [22], thus implicating it for glucose and fat homeostasis and hence, providing a mechanism for how vitamin D might contribute to overweight and/or obesity [22,23,24]. We have previously reported that vitamin D deficiency is prevalent among Ethiopian schoolchildren both in urban and rural settings accounting for 61.8% and 21.2%, respectively [25]. Other studies conducted on pregnant women and women of reproductive age group also showed that vitamin D deficiency is prevalent in Ethiopia [26,27,28]. There have been a few studies examining the prevalence of overweight and/or obesity and its associated risk factors among schoolchildren in different parts of Ethiopia [29,30,31,32]; however, none looked at whether vitamin D deficiency was associated with overweight and/or obesity among schoolchildren. In these analyses, we set out to demonstrate whether vitamin D deficiency may significantly be associated with overweight and/or obesity among schoolchildren aged 11–18 years in Ethiopia.

## 2. Methods and Materials

### 2.1. Study Design, Participants and Setup

The analyses of data set from school based cross-sectional study conducted in Adama Town (*n* = 89) and in Rural Adama *Woreda* (district) (*n* = 85) from 20 May–22 June 2013 was carried out to determine vitamin D deficiency and its association with overweight/obesity among Ethiopian schoolchildren. Data on a total of 174 schoolchildren aged 11–18 years were used for these analyses. Sample size was calculated using formula for estimating two population proportions using a power of 80%. Multi-stage stratified random sampling procedures were used to select study subjects from each study setting. Further details including sampling procedure, inclusion/exclusion criteria, protocol approval and ethics statements are published elsewhere [25].

### 2.2. Analysis of 25-Hydroxyvitamin D

After obtaining signed informed written consent from the parents/guardians and verbal assent from the children, each child had a finger pricked by trained health workers, from which free-flowing blood drops were collected on blood spot cards as per ZRT laboratory instructions [33]. At least two such usable (non-overlapping) drops were collected per subject. After air drying for at least 30 min, flaps were closed and placed in the sealed Ziploc bags with desiccant and moisture indicators, and taken to Oromia Public Health Research, Capacity Building and Quality Assurance Laboratory by principal investigator for storage at −80 °C. Samples were then sent for analysis to ZRT laboratory (Beaverton, OR, USA), within 4 days. Circulating serum 25(OH)D was analyzed from dried blood spots, using a standard liquid chromatography/tandem mass spectrometry (LC-MS/MS) assay having intra-assay and inter-assay coefficients of variation (CVs) of 8.1% to 9.2% and 12% to 13%, respectively. The ZRT Laboratory participates in DEQAS (the Vitamin D Quality Assessment Scheme), which provides control samples to ensure assay accuracy and the blood spot 25(OH)D determination is deemed equivalent to serum as stability of 25(OH)D in serum or plasma is high [33]. A cut-off for deficiency of <50 nmol/L was based on consideration of the Endocrine Society Clinical Practice for Evaluation, Treatment, and Prevention of Vitamin D Deficiency [34].

### 2.3. Anthropometric Data

Body weight, height, and triceps skinfold thickness were measured using a precision digital scale (HD-318; TANITA), portable stadiometer, and Holtain skinfold caliper, respectively. Children removed their shoes and jackets for height and weight measurements. Body Mass Index (BMI) was calculated as weight in kilogram divided by height in squared meter (kg/m^2^). The triceps skinfold thickness (TSF) was measured at the upper arm mid-point mark between acromion process of shoulder blade and olecranon process of ulna on the posterior surface of the left upper arm. Weight, height and skinfold thickness were measured 3 times and the means were used for analyses. Data for BMI were compared to new 2007 WHO references for specific age and sex using Anthroplus software and data for TSF were compared to 1995 WHO references for specific age and sex [35] as there is no reference value for TSF from more recent WHO references. Every morning and prior to each measurement, the weight scale was calibrated with a standard weight and instruments were calibrated according to the manufacturer’s recommendations.

### 2.4. Socioeconomic Index

Socioeconomic index was categorized as follows: first all study participants were asked about the ownership of fixed assets by their respective households with a score “1” given to those who own the asset and score of “0” given to those who did not own. Then, all items asked were assessed for internal consistency and showed to be reliable with a Cronbach’s alpha value of 0.82. Then, principal component analysis was used to generate wealth index from fixed assets, including ownership of household furniture, equipment, animals and other local indicators of wealth such as mills, generator, bicycle, motor cycle and beehive. Then wealth index was rank ordered into tertiles to give low, middle and high socioeconomic status.

### 2.5. Statistical Analysis

Data were checked for missing values and outliers, and analyzed using SPSS for window (SPSS Inc., version 16.1, Chicago, IL, USA). Normality of the continuous variables was checked visually using inspection of the skewness and kurtosis measures of their respective standard errors, histograms and Q-Q plots of residuals against the predicted values and box plots and using the Kolmogrov–Smirnov test. Logarithmic transformations were made as necessary for data that were not normally distributed. Mean values between groups were compared using independent sample *t*-test for normally distributed data and nonparametric tests (Mann–Whitney test and Kruskal–Wallis test) were used for data that were not normally distributed. Chi-square tests were used to compare categorical data between groups. TSF values of students were not normally distributed even after logarithmic transformations. Hence, TSF values were grouped into two (*i.e.*, TSF-for-age ≥90th percentile as “overweight and/or obese” and <90th percentile as “not overweight and/or obese”) to allow entry into the logistic regression model. Then, to determine predictors associated with overweight and/or obesity, we first carried out bivariate analyses to identify candidate variables for the multivariable logistic regression model. Second, to identify the significant independent predictors associated with overweight and/or obesity at an alpha level of 0.05, variables that had *p* < 0.25 in the bivariate analyses were entered into multivariable logistic regression model using backward elimination stepwise likelihood ratio method. All tests were two-sided and *p* < 0.05 was considered statistically significant. The results were reported as Odds Ratio (OR) and 95% Confidence Interval (CI).

## 3. Results

A total of 174 study participants from the schools in two settings, in Adama Town (89 (51.1%)) and rural kebeles (wards) of Adama Woreda (85 (48.9%)) participated in the study (response rate 98%). The median (range) age of the students was 15 (11–18) years. Higher proportions of students were females than males (56.9% *vs*. 43.1%), and in the age group 15–18 years (55.7%); most (79.9%) of them were Christian by religion. The majority of mothers and fathers had attended a formal education (69% and 55.4%, respectively). Only a small proportion of mothers and fathers were employed. Almost equal proportions of the study subjects were from households in low (34.5%) and high (35.1%) socioeconomic status (Table 1).

The overall prevalence of overweight and/or obesity was 10.3%, with 8.5% overweight and 2.3% obese using TSF-for-age. The prevalence became less (6.9%) using BMI-for-age. The prevalence of underweight was 19% using BMI. The mean and median BMI and TSF of the students were 17.9 ± 2.9 kg/m^2^ and 12 (5.2–31.2 mm), respectively. Average serum 25(OH)D for the group was 54.5 ± 15.8 nmol/L. Nearly half (42%) of the study subjects were vitamin D deficient (serum 25(OH)D < 50 nmol/L) while 49.4% were vitamin D insufficient (serum 25(OH)D 50–74.9 nmol/L). Only 8.6% of the study subjects were vitamin D sufficient (serum 25(OH)D ≥ 75 nmol/L). The deficiency is more prevalent in girls and urban students compared to their respective counterparts (51.5% *vs*. 29.3% and 61.8% *vs*. 21.2%, respectively). Students in the higher (15–18 years) age group had higher prevalence of vitamin D deficiency (49.5%) compared to those in lower (11–14 years) age group (32.5%). Vitamin D deficient children had statistically significantly (*p* = 0.003) higher BMI than children with normal vitamin D status. Serum 25(OH)D was inversely correlated with both BMI and TSF values (*r* = −0.233; *p* = 0.002 and Spearman’s rho = −0.395; *p* < 0.001, respectively).

A Mann–Whitney U test showed that median TSF was significantly higher for vitamin D deficient (serum 25(OH)D < 50 nmol/L) group compared to a group with normal vitamin D status (*p* < 0.001). Likewise, the median TSF among older students was significantly higher than their younger counter parts (*p* < 0.001). Furthermore, females had significantly higher median TSF value than males (*p* < 0.001). It was also observed that the median TSF values differed significantly according to the following selected variables, respectively: students who had usual outdoor activities for less than 30 min both on school days and on weekend days had higher median TSF value than those who had usual outdoor activities for greater than 30 min, students from mothers who have formal education had higher TSF value than students from mothers who have no formal education, and students from urban setting had higher TSF value than those from rural setting. However, there was no statistically significant difference between the median TSF values of respondents according to the occupation of both their fathers and mothers and also according to education status of their fathers. A Kruskal–Wallis test showed that median TSF values of respondents differ significantly among low, middle and high socioeconomic groups of the study subjects (Table 2).

In bivariate logistic regression analyses, all variables except paternal education and paternal occupation had *p*-values of less than 0.25 and thus, entered into multivariable logistic regression model using backward elimination stepwise likelihood ratio method to identify independent predictors significantly associated with overweight and/or obesity. Accordingly, vitamin D deficiency, higher ager age group, female sex and residence of students, and their mothers’ occupation of being employed and their households’ high and middle socioeconomic status were independent predictors significantly associated with overweight and/or obesity after controlling for potential confounders using multivariable logistic regression model (Table 3).

Vitamin D deficiency was significantly associated with overweight and/or obesity in schoolchildren after controlling for possible confounding factors using multivariable logistic regression model. Vitamin D deficient students were about 4.6 times as likely to be overweight and/or obese compared to those students who had normal vitamin D status (AOR = 4.59 (1.11, 18.91)). In addition, some socio-economic and demographic factors were found to have statistically significant association with overweight and/or obesity. Older children were about 6.7 times more likely to be overweight and/or obese compared to younger ones (AOR = 6.66 (1.46, 30.29)), while females were 7.72 times more likely to be overweight and/or obese than males (AOR = 7.72 (1.52, 39.30)). Students whose mothers were employed had 5.26 times more odds of being overweight and/or obese compared to their counter parts (AOR = 5.26 (1.09, 25.36)). The respective odds of being overweight and/or obese among students from high and middle socioeconomic status households were 10.9 and 17.9 times higher compared to students from low socioeconomic status households (AOR = 10.90 (2.14, 55.47) and AOR = 17.89 (1.12, 87.18), respectively). Similarly, students living in urban settings were 5.3 times more likely to be overweight and/or obese compared to those living in rural setting when controlled for age groups and vitamin D status of students and their households’ socioeconomic status (AOR = 5.29 (1.45, 19.36)).

## 4. Discussion

The present study demonstrates that vitamin D deficiency was significantly associated with overweight and/or obesity determined using TSF among schoolchildren in Ethiopia. There is evidence that skinfold thickness measurements are better predictors of body fat percentage than BMI in children and adolescents [36] as the use of BMI to identify overweight children at risk for metabolic disorders has limitations [37]. There is evidence from cell culture and animal model studies that adipose tissue has both the vitamin D receptor (VDR) and the ability to synthesize 1,25-dihydroxyvitamin D_3_, the bioactive vitamin D metabolite, and that vitamin D may regulate adipose tissue mass, differentiation and metabolism in ways that might contribute to overweight and/or obesity possibly by effects on lipogenesis and/or adipogenesis [38,39,40,41,42,43].

Although the exact biological mechanisms by which vitamin D may influence the various metabolic disorders are still under investigation, research findings from different epidemiological studies indicate that inadequate vitamin D has been found to be a predictor significantly associated with overweight and/or obesity and other components of metabolic syndrome including raised plasma glucose concentration and insulin resistance [44]. A number of studies have shown that vitamin D status is significantly associated with overweight and/obesity in children and adolescents. A cross-sectional study conducted on the nationally representative sample of Korean adolescents indicated that being vitamin D deficient (25(OH)D level < 50 nmol/L) appeared to be associated with higher risk for overweight or obesity, even after adjusting for confounders [45]. Moreover, Lee *et al*. [46] reported that serum 25(OH)D concentrations had significant inverse association with adiposity indices in preadolescent children in Korea. In this study, for a unit increase in serum 25(OH)D concentration waist circumference (WC), BMI and body fat mass (BFM) were reduced by 28%, 10% and 12%, respectively; however, body fat percentage (BFP) and TSF showed only marginal boundary for the association. Furthermore, this study showed that the overall risk of overweight was reduced by 13% per unit increase of 25(OH)D even after adjusting for relevant confounding factors. The risk of obesity also decreased with a unit increase in the 25(OH)D level, but *p-*values for these associations showed borderline significance [46]. These findings support our data which showed that for a unit increase in serum 25(OH)D level, the risk of overweight and/or obesity is reduced by 5.6% when adjusting for confounders using multivariable logistic regression model suggesting a possible protective role of vitamin D against overweight and/or obesity in our study. Similarly, a cross-sectional study by Zhang *et al*. [47] among Chinese schoolchildren showed that the vitamin D deficiency group had significantly higher body weight, BMI, waist circumference and % body fat (PBF), all of which are proxy indicators of overweight and/or obesity. Our study findings are in line with these findings demonstrating that vitamin D deficient subjects had significantly higher mean BMI and higher median TSF than their counterparts with normal vitamin D status.

In addition, a case control study undertaken on overweight/obese Caucasian children and adolescents and their controls of normal weight in the Rome area revealed that there were multivariable-adjusted significant associations between low serum 25(OH)D levels and obesity and other cardiometabolic risk factors [44].

Prospective epidemiological studies in human subjects also reported vitamin D deficiency is significantly associated with overweight and/or obesity. For instance, Gilbert-Diamond *et al*. [48] in their three-year prospective study among schoolchildren from Colombia concluded that low vitamin D status (25(OH)D levels < 50 nmol/L) was inversely associated with the development of adiposity. This study indicated that vitamin D deficient children had 0.1 kg/m^2^, 0.03 and 0.8 cm significantly greater yearly change in BMI, subscapular-to-triceps skinfold thickness ratio and waist circumference, respectively, than did vitamin D sufficient children suggesting vitamin D deficiency is associated with adiposity. A similar findings were reported in follow-up study of younger children in Korea where those children with the lowest quartile of 25(OH)D levels had the highest serum triacylglycerol levels that might be due to higher adiposity [49]. Similarly, a longitudinal study on older Norwegians adult population revealed that low serum 25(OH)D levels (<50 nmol/L) were associated with a significantly increased risk for incident obesity during a follow-up period [50].

On the other hand, evidences show that overweight/obesity is associated with vitamin D deficiency. Indeed, we have previously reported that overweight/obese children were found to have lower serum 25(OH)D than non-overweight/obese children [25]. This might be due to either volumetric dilution of vitamin D in the large fat mass or its increased uptake by adipose tissue in overweight/obese subjects [51,52,53]. In our study it was observed that students in high and middle socioeconomic groups were more likely to be overweight/obese. There is evidence that overweight/obesity in children is generally considered to have a positive association with socioeconomic status in developing countries [20]. Similarly, being in higher age group, urban residence and female sex were significantly associated with overweight/obesity. This might be due to reduced physical activities and increased sedentary life style behaviors with age and in urban students and in girls [54]. Furthermore, students whose mothers were employed had significantly increased risk of being overweight/obese. This might be explained by employed mothers could have inadequate time to supervise their children resulting in consumption of more unhealthy foods and less healthy foods of the children that could increase this risk [55]. The other possible reason for higher level of overweight/obesity among urban children could be due to their higher consumption of animal source foods that could be rich in fats as discussed in our previous study [25].

This study is the first one to look at the associations between vitamin D status and risk of overweight and/or obesity in Ethiopian schoolchildren. In addition, it might be among few studies that assessed overweight/obesity among school children using BMI-for-age and TSF-for-age. As childhood overweight/obesity is highly linked to adulthood obesity, the findings have wider implication on designing of prevention strategies for the emerging problem of adult chronic non-communicable diseases and associated morbidity and mortality in Ethiopia.

However, our study has some limitations. As a cross-sectional study, the associations it demonstrated do not imply a causal relationship between serum 25(OH)D concentrations and risk of overweight and/or obesity in our study subjects. In addition, the study may be confounded by a lack of data on some relevant variables such as information on total energy intake, more detailed physical activity levels, and dietary vitamin D intake such as egg yolk, fish and fish liver oil, meat and yeast fermented cereal based foods that could be available and accessible for consumption by the study subjects.

## 5. Conclusions

In conclusion, vitamin D deficiency was found to be an independent predictor significantly associated with overweight and/or obesity among Ethiopian schoolchildren. The results imply the need for behavior change communication on the importance of exposure to sunlight to produce adequate vitamin D in Ethiopian schoolchildren to curb this emerging health problem of overweight/obesity and its associated long term health consequences following economic growth and globalization in Ethiopia. As the present study only highlighted the association between vitamin D deficiency and overweight/obesity in our study subjects, prospective studies and randomized controlled trials are required to establish causality.

## Figures and Tables

**Table 1:** Characteristics of schoolchildren in Central Ethiopia.

Characteristics (*n* = 174)		Frequency (%)	
Sex	Male	75 (43.1)	
	Female	99 (56.9)	
Age groups	11–14	77 (44.3)	
	15–18	97 (55.7)	
Residence	Urban	89 (51.1)	
	Rural	85 (48.9)	
Religion	Christians	139 (79.9)	
	Muslims	35 (20.1)	
Educational status (Father)	No formal education	54 (31)	
	Formal education	120 (69)	
Educational status (Mother)	No formal education	77 (44.3)	
	Formal education	97 (55.7)	
Occupation (Father)	Employed	50 (28.7)	
	Self employed/Unemployed	124 (71.3)	
Occupation (Mother)	Employed	26 (14.9)	
	Self employed/Unemployed	148 (85.1)	
Socioeconomic status	Low	60 (34.5)	
	Medium	53 (30.5)	
	High	61 (35.1)	
WHO BMI-for-age percentile	Underweight	33 (19)	
	Normal	129 (74.1)	
	Overweight and/or obese	12 (6.9)	
WHO TSF-for-age percentile	Overweight and/or obese	18 (10.3)	
	Overweight and/or obese	156 (89.7)	
Serum 25(OH)D	Deficient (<50 nmol/L)	73 (42)	
	Insufficient (50–74.9 nmol/L)	86 (49.4)	
	Sufficient (≥75 nmol/L)	15 (8.6)	
Mean BMI	17.9 ± 2.9 kg/m^2^		
Median TSF	12 (5.2–31.2) mm		
Mean serum 25(OH)D	54.5 ± 15.8 nmol/L		
Difference in mean BMI	Deficient vitamin D status ^e^	18.6 ± 3.2	*p* = 0.003
	Normal vitamin D status	17.3 ± 2.5	

^e^ Definition of vitamin D status (vitamin D deficiency = serum 25(OH)D < 50 nmol/L and normal = serum 25(OH)D ≥ 50 nmol/L).

**Table 2:** Differences in median TSF values of Ethiopian schoolchildren according to some study variables *.

Variable (*n* = 174)	Frequency	Median TSF Value	*p*-Value
Vitamin D status			
Deficient	73	16 (5.2, 31.2)	*p* < 0.001
Normal	101	10 (5.6, 29)	
Sex			
Male	75	9 (5.2, 21.5)	*p* < 0.001
Female	99	16 (5.6, 31.2)	
Age groups			
11–14	77	10.3 (5.2, 24.7)	*p* < 0.001
15–18	97	13.5 (5.6, 31.2)	
Maternal education			
Formal education	35	12 (6, 29.8)	*p =* 0.008
No formal education	139	11 (5.2, 31.2)	
Maternal occupation			
Employed	26	13.5 (5.6, 29.8)	*p* = 0.670
Unemployed	148	11.7 (5.2, 31.2)	
Paternal education			
Had formal education	54	12 (5.2, 29.8)	*p* = 0.987
Had no formal education	120	11 (5.6, 31.2)	
Paternal occupation			
Employed	50	12 (5.6, 29.8)	*p* = 0.736
Unemployed	124	11.6 (5.2,31.2)	
Daily outdoor activity on school days			
<30 min	33	20 (6, 31)	*p* < 0.001
≥30 min	141	11 (5.2, 31.2)	
Daily outdoor activity on weekend days			
<30 min	43	19 (8, 31.2)	*p* < 0.001
≥30 min	141	10.3 (5.2, 28.8)	
Residence			
Urban	89	14 (6, 31.2)	*p* = 0.048
Rural	85	10 (5.2,28.8)	
Socioeconomic status			
Low	60	9.7 (5.2, 28.8)	*p* < 0.001
Middle	53	13 (5.6, 31.2)	
High	61	13.2 (6, 29.8)	

* Values are in median (range); TSF = triceps skin fold thickness.

**Table 3:** Predictors associated with overweight and/or obesity in multivariable logistic regression analysis for Ethiopian schoolchildren.

Variables (*n* = 174)		Overweight and/or Obese		COR (95% CI)	AOR (95% CI)
		Yes	No		
		Number (%)	Number (%)		
Vitamin D status ^1^					
	Deficient	14 (77.8)	59 (37.8)	5.75 (1.81, 18.31)	4.59 (1.11, 18.91)
	Normal	4 (22.2)	97 (62.2)	Referent	Referent
Serum 25(OH)D level ^1^				0.945 (0.91, 0.98)	0.946 (0.89, 0.99) ^µ^
Residence *					
	Urban	15 (83.3)	74 (47.4)	5.54 (1.54, 19.90)	5.29 (1.45, 19.36)
	Rural	3 (16.7)	82 (52.6)	Referent	Referent
Gender					
	Male	2 (11.1)	73 (46.8)	Referent	Referent
	Female	16 (88.9)	83 (53.2)	7.04 (1.57, 31.64)	7.72 (1.52, 39.30)
Age groups					
	11–14	3 (16.7)	74 (47.4)	Referent	Referent
	15–18	15 (83.3)	82 (52.6)	4.51 (1.26, 16.21)	6.66 (1.46, 30.29)
Education (Father)					
	Formal education	14 (77.9)	106 (67.9)	Referent	Referent
	No formal education	50 (32.1)	4 (22.1)	0.61 (0.19, 1.93)	**
Education (Mother)					
	Formal education	13 (72.2)	84 (53.8)	Referent	Referent
	No formal education	5 (27.8)	72 (46.2)	0.45 (0.15, 1.32)	1.31 (0.28, 6.05)
Occupation (Father)					
	Employed	6 (33.3)	44 (28.2)		**
	Unemployed	12 (66.7)	112 (71.8)	Referent	Referent
Occupation (Mother)					
	Employed	5 (27.8)	21 (13.5)	2.473 (0.79, 7.65)	5.26 (1.09, 25.36)
	Unemployed	13 (72.2)	135 (86.5)	Referent	Referent
Socioeconomic status					
	Low	3 (6.7)	57 (36.6)	Referent	Referent
	Middle	8 (44.4)	45 (28.8)	0.41 (0.10,1.65)	17.89 (1.12, 87.18)
	High	7 (38.9)	54 (34.6)	1.37 (0.46, 4.07)	10.90 (2.14, 55.47)
Daily outdoor activity on school days					
	<30 min	8 (44.4)	25 (16.0)	4.19 (1.51, 11.67)	0.89 (0.21, 3.77)
	≥30 min	10 (55.6)	131 (84.0)	Referent	Referent
Daily outdoor activity on weekend days					
	<30 min	11 (61.1)	32 (20.5)	6.09 (2.19, 16.96)	2.33 (0.46, 11.79)
	≥30 min	7 (38.9)	124 (79.5)	Referent	Referent

^1^ Estimation of parameters was done by entering both forms of the variable into the model separately to avoid collinearity between them; COR = Crude Odds Ratio; AOR = Adjusted Odds Ratio; CI = confidence interval; * Controlled for age groups, vitamin D status and socioeconomic status; ** No AOR reported as both paternal education and occupation were not entered into the multivariable logistic model; ^µ^ β coefficient for serum 25(OH)D = −0.056.
